# Supplementary material for: Uncovering bridging diseases in complex multimorbidity pathways: A network science approach
Source: PLoS One. 2025 May 7;20(5):e0323208. doi: 10.1371/journal.pone.0323208 (PMC12058136; doi:10.1371/journal.pone.0323208)
Supplement: Attachment — Submitted filename: Response to reviewers_v2.docx [file pone.0323208.s002.docx]

**Response to reviewers**

Dear editor, Dear reviewers,

We sincerely appreciate the time and effort you have invested in reviewing our manuscript titled “Uncovering Bridging Diseases in Complex Multimorbidity Pathways: A Network Science Approach.” Your accurate comments and constructive feedback have helped us refine and improve the general clarity, methodological rigor, and overall quality of our manuscript. Below, we provide a detailed response to each of your comments and outline the corresponding revisions made to the manuscript.

Best regards,

The Co-authors

**Response to reviewer #1:**

**1. Abstract:**

**Comment (C): The introduction of the abstract should provide a clearer context and set the stage for the study’s objectives.**

Response (R): We have revised the abstract to include a brief introduction on multimorbidity profiles and their significance in health research. This provides a better context before presenting the methodology and findings.

**2. Presentation and Clarity:**

**C: Minor rephrasing for clarity and grammatical errors should be addressed.**

R: We conducted a thorough proofreading of the manuscript, and we also have included subsections structure for better readability and clarity.

**3. Gender-Specific Health Inequalities:**

**C: Expand on how findings can inform practical healthcare strategies for both sexes.**

R: We have elaborated in the discussion section on how our findings can be used to inform gender-specific healthcare interventions.

**4. Future Directions:**

**C: Consider suggesting a longitudinal approach to understand disease trajectories in multimorbidity progression.**

R: We have added a section in the discussion suggesting future research directions, emphasizing the need for longitudinal studies to track multimorbidity evolution over time.

**Response to reviewer #2:**

**1. Clarity Issues:**

**C: Some sentences need rewording for clarity.**

R: As recommended, we revised sentences throughout the manuscript to improve clarity and conciseness.

**2. Methods:**

**C: Justify the use of an estimation error of ±2.5 units when a 5% margin is more common.**

R: Thanks for this observation. We have provided an explanation in the methods section for choosing ±2.5 units, emphasizing the need for higher precision in estimating multimorbidity patterns.

**C: Explain why selection criteria were not applied after determining a minimum sample size of 2,200.**

R: We clarified that the full sample was initially considered (to have a representative sample of people in this area, including also 50+ people without multimorbidity), and selection criteria were applied subsequently to focus exclusively on individuals with multimorbidity.

**C: Clarify the calculation of response rate.**

R: We have included a more detailed explanation of how the response rate was calculated, addressing the difference between the total surveyed individuals and those included in the final analysis.

**C: Improve the organization of sample size, inclusion/exclusion criteria, and data collection procedures.**

R: These components have been reorganized under a separate subheading to enhance the readability and logical flow of sample size calculation and selection criteria.

**C: Elaborate on how conditional independence assumptions were met.**

R: Thanks for this observation. We have explained in the methods section that “our implementation of mixed graphical models (MGM) incorporates LASSO regularization to enforce sparsity, thereby ensuring that only the strongest partial correlations remain in the network structure. This approach improves interpretability by reducing false associations and capturing the most meaningful disease relationships […] in terms of conditional independence, MGMs use the structure of a Markov random field to represent conditional relationships between variables. Formally, the model ensures that a variable is conditionally independent of the rest of the variables given its neighborhood in the network.”

Thus, our methodology not only ensures a more accurate representation of multimorbidity structures but also strengthens the validity of inferred relationships by addressing the issues of indirect associations and multicollinearity, which are common limitations in traditional regression-based approaches.

**C: Provide more details on how disease relationships are represented in the graph.**

R: We expanded the methods section to describe the parameterization process and how disease associations were captured using the mixed graphical model (MGM).

**C: Describe the logistic regression approach in greater detail.**

R: We added a more comprehensive description of our logistic regression approach.

**3. Results:**

**C: Organize results into subheadings for better clarity.**

R: We restructured the results section with subheadings: (a) Disease prevalence Estimates, (b) Detecting multimorbidity patterns, (c) Identifying bridging diseases, and (d) Association between bridging diseases and multimorbidity patterns, making it easier for readers to navigate the results. Subsections have also been incorporated into methods and discussion sections.

We sincerely appreciate the valuable feedback provided by the reviewers. These revisions have strengthened our manuscript, making it more precise, structured, and impactful. We hope the changes address all concerns and look forward to your further feedback.

Best regards,

The Co-authors
